# Supplementary material for: miR-182-5p promotes hepatocyte-stellate cell crosstalk to facilitate liver regeneration
Source: Commun Biol. 2022 Aug 1;5:771. doi: 10.1038/s42003-022-03714-0 (PMC9343643; doi:10.1038/s42003-022-03714-0)
Supplement: Supplementary file 2 — Supplementary Material [file 42003_2022_3714_MOESM2_ESM.pdf]

Supplementary Information

miR-182-5p Promotes Hepatocyte-Stellate Cell Crosstalk to Facilitate Liver Regeneration

Ting Xiao[1,#], Wen Meng [1,#,*], Zhangliu Jin[1,2], Jing Wang[1], Jiangming Deng[1], Jie Wen[1], Bilian Liu[1], Meilian Liu[1,3], Juli Bai[1,4] and Feng Liu[1*]

[1]National Clinical Research Center for Metabolic Diseases, Metabolic Syndrome Research Center, Key Laboratory of Diabetes Immunology, Ministry of Education, and Department of Metabolism and Endocrinology, The Second Xiangya Hospital of Central South University, Changsha 410011, Hunan, China.
[2]Department of Biliopancreatic Surgery and Bariatric Surgery, The Second Xiangya Hospital of Central South University, Changsha 410011, Hunan, China.
[3]Department of Biochemistry and Molecular Biology, University of New Mexico Health Sciences Center, Albuquerque, NM
[4]Department of Pharmacology, University of Texas Health Science Center at San Antonio, San Antonio, TX

*Corresponding authors. Addresses: National Clinical Research Center for Metabolic Diseases, Metabolic Syndrome Research Center, and Department of Metabolism and Endocrinology, The Second Xiangya Hospital of Central South University, 139 Middle Renmin Road, Changsha 410011, Hunan, China (Feng Liu ; Wen Meng).
E-mail addresses: liuf001@csu.edu.cn (Feng Liu), 122501006@csu.edu.cn (Wen Meng).

# These authors contribute equally to the work.
* Corresponding author.

Running title: Hepatic miR-182-5p in Liver Regeneration Regulation

Keywords: miR-182-5p; Liver regeneration; Stellate cell; Cholic acid; Hedgehog signaling

Word Count: 6393 words.
Number of Figures: 7
Number of Tables: 0

Supplemental figures

Figure S1

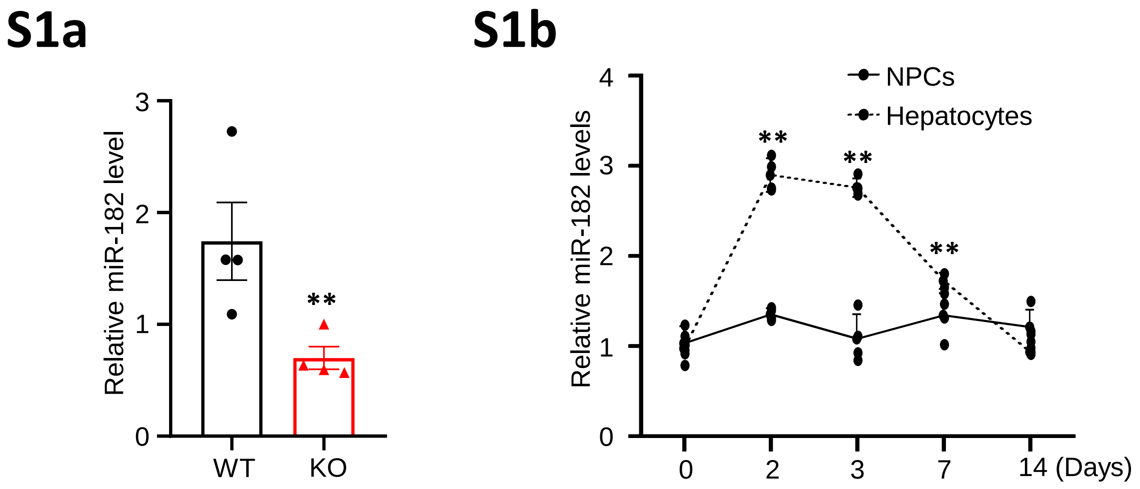

Fig. S1. Hepatocellular miR-182-5p is upregulated during mouse liver regeneration. (a) qRT-PCR analyses of miR-182-5p expression level in liver of miR-182 KO and WT mice (n=4/group). **(b)** qRT-PCR analyses of miR-182-5p expression level in primary hepatocytes and NPCs of isolated from WT mice after PH (n=4/group). Error bars in all experiments represent SEM; Significance was determined by unpaired 2-tailed Student's *t* test and by one-way ANOVA. ***P* < 0.01.

Figure S2

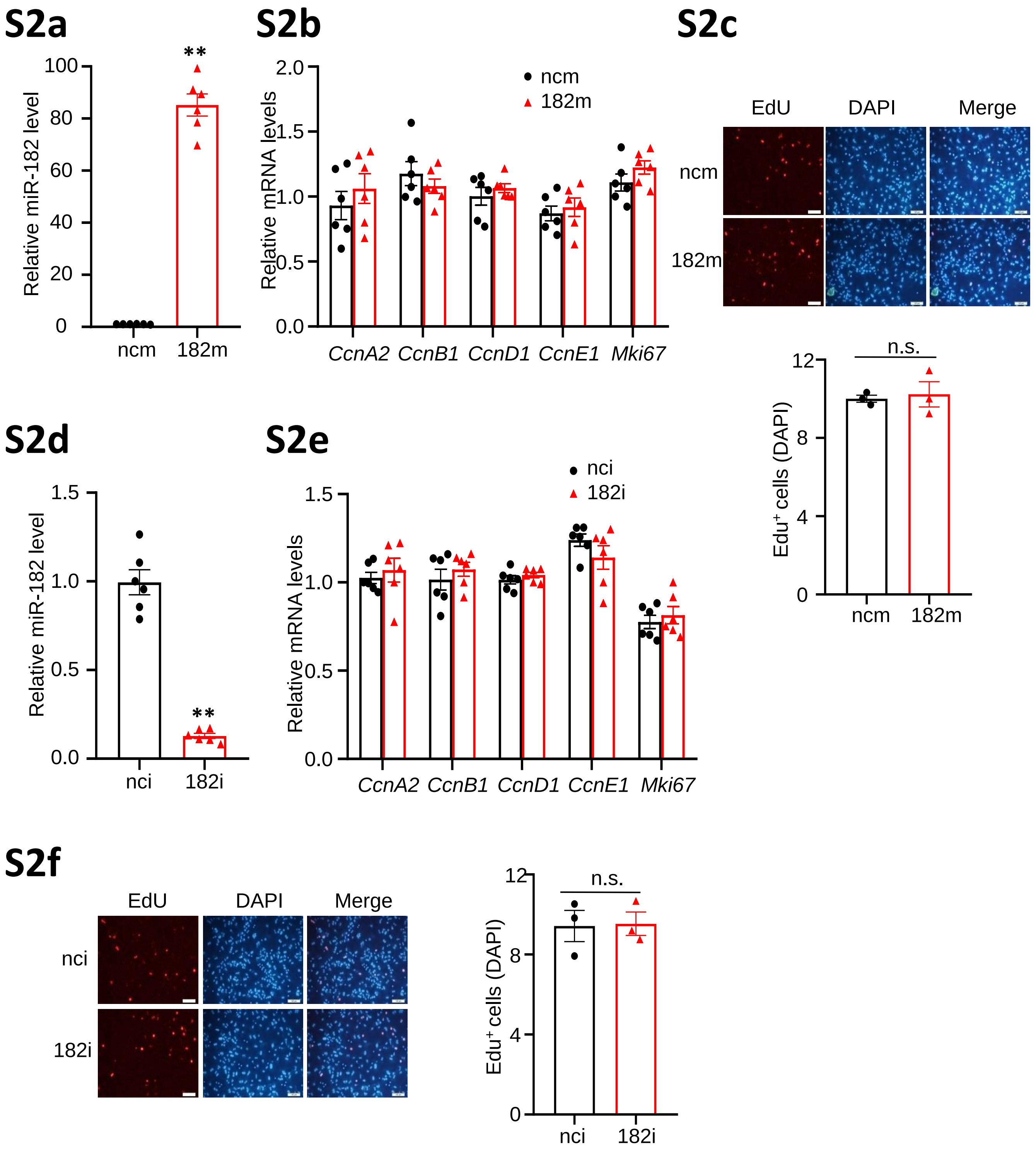

Fig. S2. miR-182-5p is insufficient to promote proliferation in cultured hepatocytes. Primary hepatocytes were treated with 30 ng/mL EGF to induce hepatocyte proliferation. miR-182-5p mimic (182m) or its negative control (ncm) were overexpressed in primary hepatocytes isolated from C57BL/6J mice. miR-182-5p level **(a)** and the cyclin gene levels **(b)** were determined by qRT-PCR (n=6/group). **(c)** EdU immunostaining of primary hepatocytes transfected with miR-182-5p mimic (182m) or its negative control (ncm) (n=3/group; scale bar: 100µm). Mouse primary hepatocytes from C57BL/6J mice were transfected with miR-182-5p inhibitor (182i) or its negative control (nci), and then the cells were treated with EGF (30ng/ml) for 24h. miR-182-5p level **(d)** and the cyclin gene levels **(e)** were determined by qRT-PCR(n=6/group). **(f)** EdU immunostaining of primary hepatocytes transfected with miR-182-5p inhibitor (182i) or its negative control (nci) (n=3/group; scale bar: 100µm). Error bars in all experiments represent SEM; Significance was determined by unpaired 2-tailed Student's *t* test. ***P* < 0.01; n.s.: not significant.

Figure S3

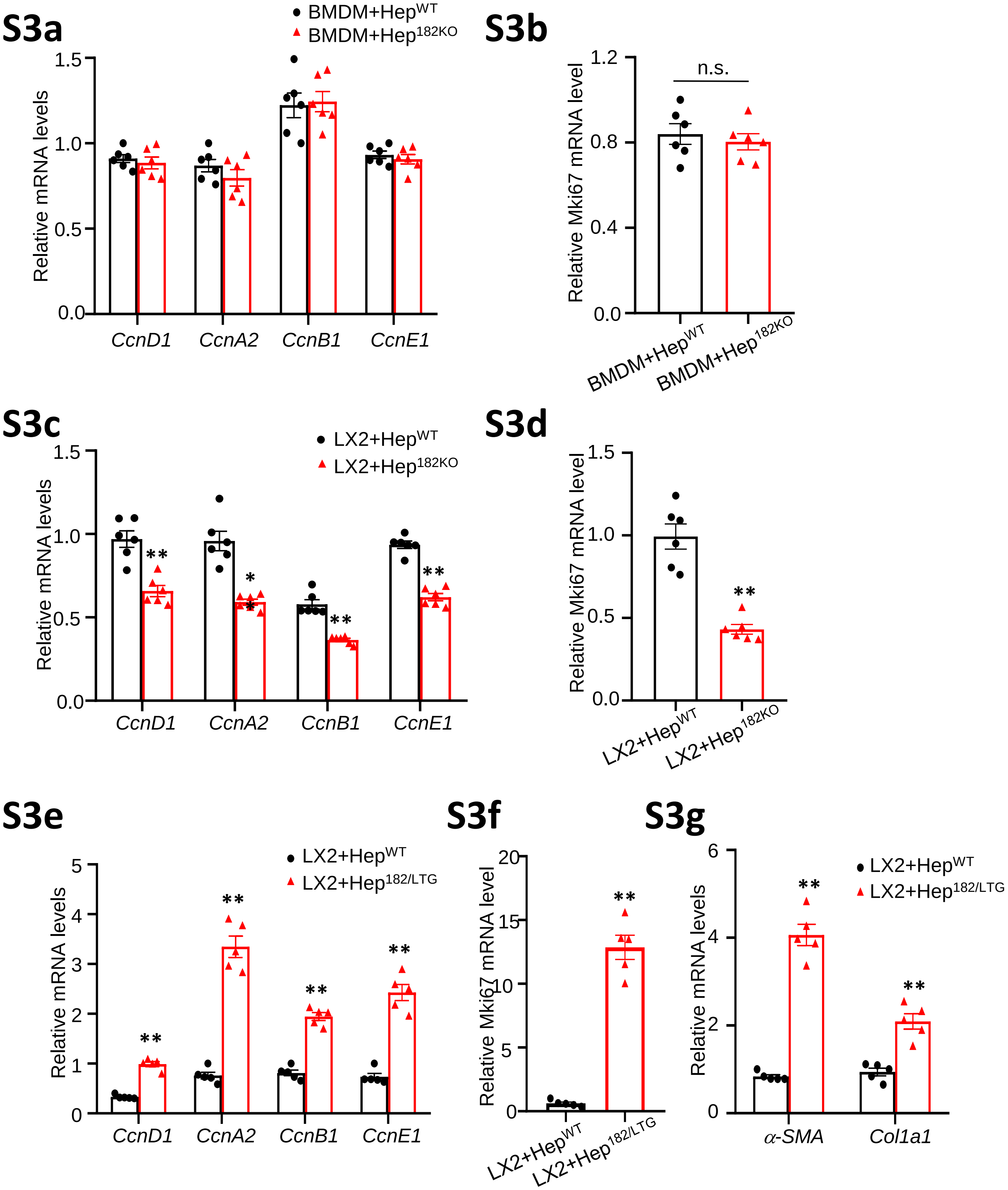

Fig. S3. miR-182-5p regulates mouse hepatocyte proliferation via a stellate cell-dependent mechanism. Primary hepatocytes from miR-182-5p KO (Hep^{182KO}) and WT mice (Hep^{WT}) co-cultured with BMDM. qRT-PCR analyses of *cyclin* genes **(a)** and *Mki67* gene **(b)** expression in primary hepatocytes (n=6/group). qRT-PCR analyses of *cyclin* genes **(c)** and *Mki67* **(d)** expression in primary hepatocytes from miR-182-5p KO mice ($Hep182^{KO}$) and their control mice (Hep^{WT}) co-cultured with HSCs (LX2 cells) in the presence of EGF (n=6/group). Primary hepatocytes from miR-182-5p TG mice ($Hep^{182/LTG}$) and their control mice (Hep^{WT}) co-cultured with LX2. qRT-PCR analyses of cyclin genes **(e)** and *Mki67* gene **(f)** expression in primary hepatocytes. **(g)** qRT-PCR analyses of HSC activation marker genes expression in LX2 (n=5/group). Error bars in all experiments represent SEM; Significance was determined by unpaired 2-tailed Student's *t* test.**$P < 0.01$; n.s.: not significant.

Figure S4

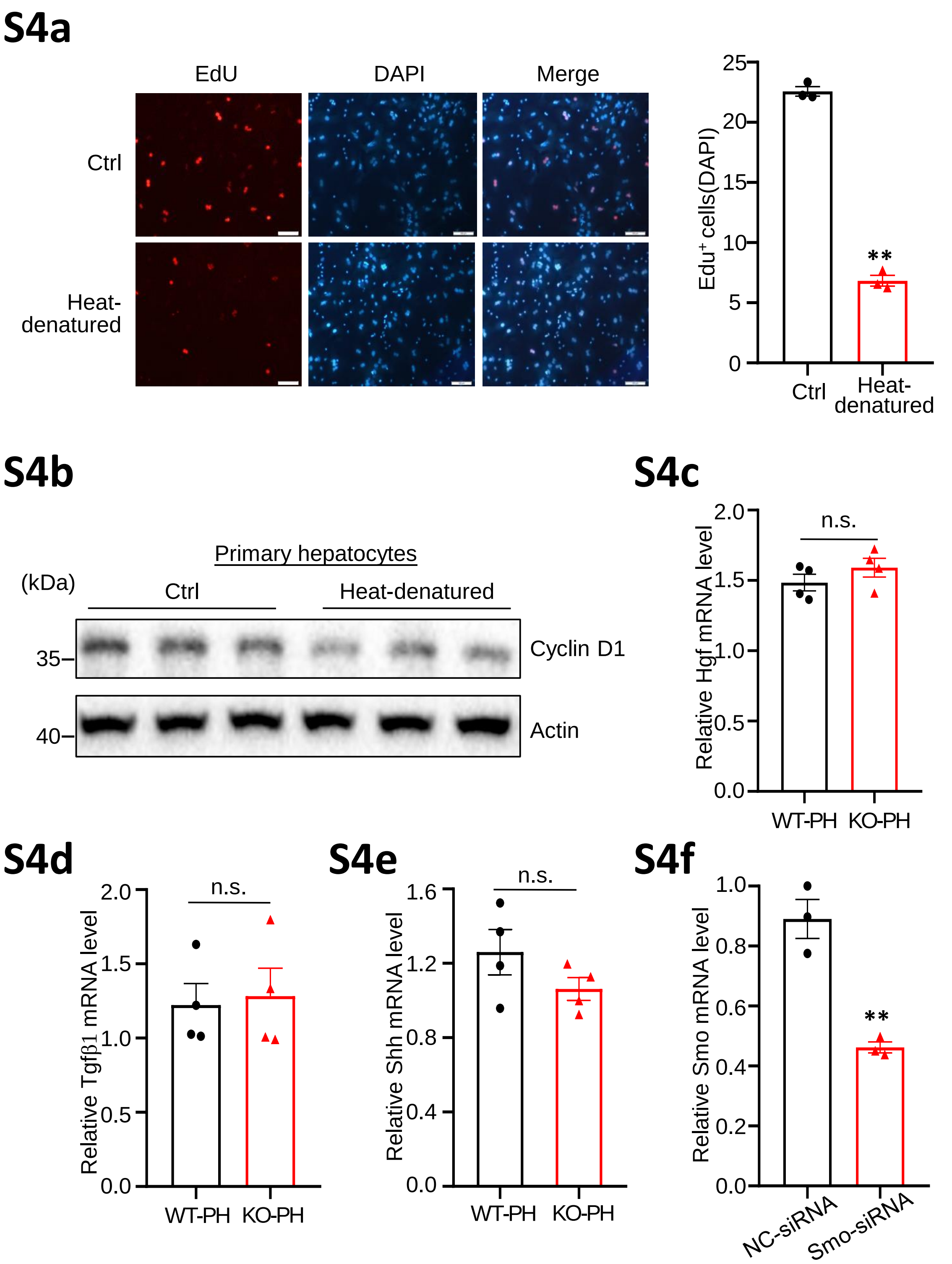

Fig. S4. miR-182-5p promotes hepatocyte proliferation by stellate cell-dependent activation of hedgehog signaling. Primary hepatocytes were treated with heat-denatured or control conditioned (Ctrl) medium of stellate cells co-cultured with miR-182-5p-overexpressed hepatocytes for 24h. **(a)** EdU immunostaining of primary hepatocytes (n=3/group). **(b)** Western Blot analysis of Cyclin D1 levels in primary hepatocytes. qRT-PCR analyses of *Hgf* **(c)**、*Tgfβ1* **(d)** *and Shh* **(e)** gene expression in the liver of miR-182-5p KO and WT mice (3d after PH; n=4/group). Primary hepatocytes were treated with conditioned medium of stellate cells co-cultured with miR-182-5p-overexpressed hepatocytes treated with Smo siRNA or control siRNA (NC-siRNA). **(f)** qRT-PCR analyses of *Smo* gene expression in primary hepatocytes (n=3/group). Error bars in all experiments represent SEM; Significance was determined by unpaired 2-tailed Student's *t* test. $^{**}P < 0.01$; n.s.: not significant.

Figure S5

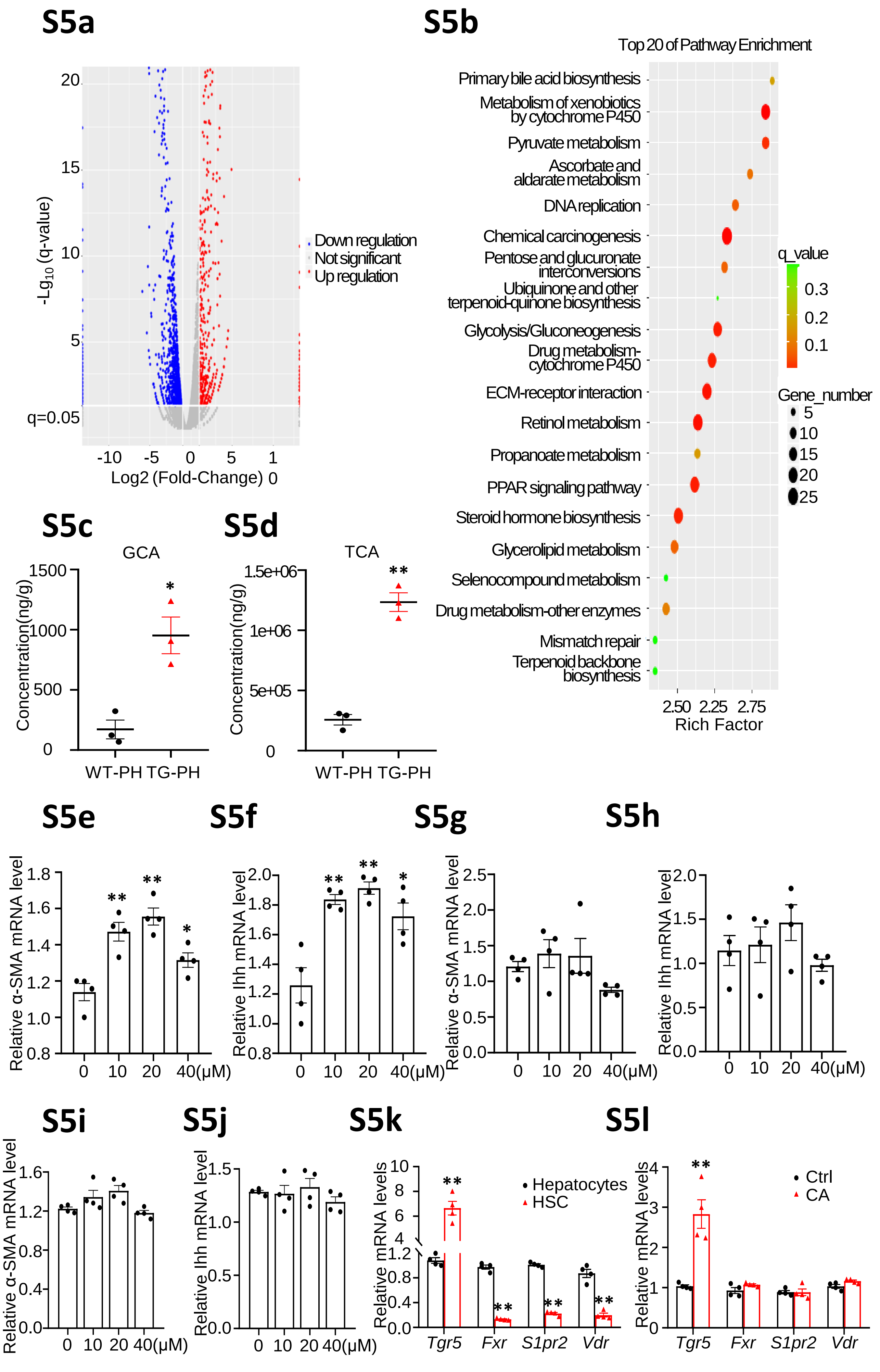
S5a
-Lg10 (q-value)
20
15
10
5
q=0.05
-10 -5 0 5 1
Log2 (Fold-Change) 0
Down regulation
Not significant
Up regulation
S5b
Top 20 of Pathway Enrichment
Primary bile acid biosynthesis
Metabolism of xenobiotics by cytochrome P450
Pyruvate metabolism
Ascorbate and aldarate metabolism
DNA replication
Chemical carcinogenesis
Pentose and glucuronate interconversions
Ubiquinone and other terpenoid-quinone biosynthesis
Glycolysis/Gluconeogenesis
Drug metabolism-cytochrome P450
ECM-receptor interaction
Retinol metabolism
Propanoate metabolism
PPAR signaling pathway
Steroid hormone biosynthesis
Glycerolipid metabolism
Selenocompound metabolism
Drug metabolism-other enzymes
Mismatch repair
Terpenoid backbone biosynthesis
2.50 2.25 2.75
Rich Factor
q_value
0.3
0.2
0.1
Gene_number
5
10
15
20
25
S5c
GCA
Concentration(ng/g)
1500
1000
500
0
*
WT-PH TG-PH
S5d
TCA
Concentration(ng/g)
1.5e+06
1e+06
5e+05
0
**
WT-PH TG-PH
S5e
Relative α-SMA mRNA level
1.8 1.6 1.4 1.2 1.0 0.8
** ** *
0 10 20 40(μM)
S5f
Relative Ihh mRNA level
2.0 1.8 1.6 1.4 1.2 1.0 0.8
** ** *
0 10 20 40(μM)
S5g
Relative α-SMA mRNA level
2.5 2.0 1.5 1.0 0.5 0.0
0 10 20 40(μM)
S5h
Relative Ihh mRNA level
2.0 1.5 1.0 0.5 0.0
0 10 20 40(μM)
S5i
Relative α-SMA mRNA level
1.6 1.2 0.8 0.4 0.0
0 10 20 40(μM)
S5j
Relative Ihh mRNA level
1.5 1.0 0.5 0.0
0 10 20 40(μM)
S5k
Relative mRNA levels
10 8 6 4 1.2 0.8 0.4 0.0
Hepatocytes
HSC
** ** ** **
Tgr5 Fxr S1pr2 Vdr
S5l
Relative mRNA levels
4 3 2 1 0
Ctrl
CA
**
Tgr5 Fxr S1pr2 Vdr

Fig. S5. miR-182-5p promotes hepatocyte proliferation by enhancing cholic acid (CA)-mediated activation of HSCs. (a) Volcano plot of the DEGs. Blue indicates down-regulated genes and red indicates upregulated genes. **(b)** Bubble plot of KEGG pathways of differentially expressed gene (DEGs). Higher and lower enrichment are indicated by red and green color, respectively. The point size indicates the number of DEGs enriched in a certain pathway. Higher rich factors indicate more significant enrichment. LC/MS analyses of the GCA **(c)** and TCA **(d)** levels in liver of the miR-182-5p TG mice and their control mice (WT) at day 3 after 2/3 PH (n=3/group). qRT-PCR analyses of *α-SMA* and *Ihh* genes expression in LX2 by CA **(e, f),** GCA **(g, h)** or TCA **(i, j)** treatment (n=4/group **(k)** qRT-PCR analyses of CA receptors genes expression in primary HSCs and primary hepatocytes isolated from WT mice. **(l)** qRT-PCR analyses of CA receptors genes expression in primary HSCs by CA treatment (n=4/group). Error bars in all experiments represent SEM; Significance was determined by unpaired 2-tailed Student's *t* test and by one-way ANOVA. *$P < 0.05$; **$P < 0.01$.

Figure S6

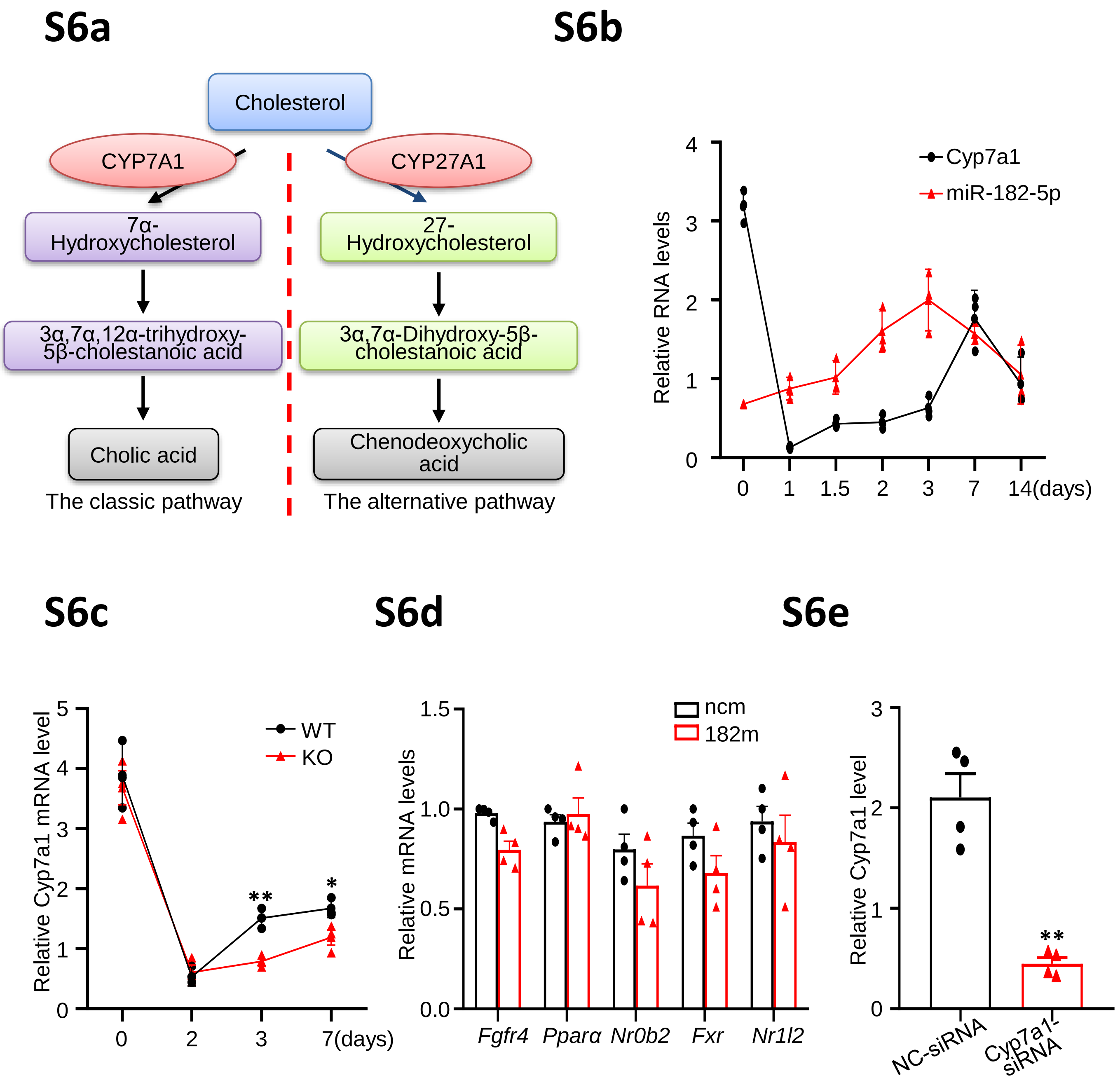

Fig. S6. Hepatic miR-182-5p targets Cyp7a1 to promote hepatocyte proliferation. (a) A schematic diagram of classic and alternative bile acid (BA) synthesis pathway. miR-182-5p mimic (182m) or its negative control (ncm) were overexpressed in primary hepatocytes from C57BL/6J mice. **(b)** qRT-PCR analyses of *Cyp7a1* and miR-182-5p expression in liver after PH. **(c)** qRT-PCR analyses of *Cyp7a1* gene expression in liver of WT and KO mice after PH. **(d)** The mRNA levels of these negatively regulators of *Cyp7a1* gene were determined by qRT-PCR(n=4/group). **(e)** qRT-PCR analyses of *Cyp7a1* gene expression in primary hepatocytes (n=4/group). Error bars in all experiments represent SEM. Significance was determined by unpaired 2-tailed Student's *t* test.

The unprocessed gel blot images with size markers are provided in Supplementary Fig. 7

Figure S7

Figure 1d

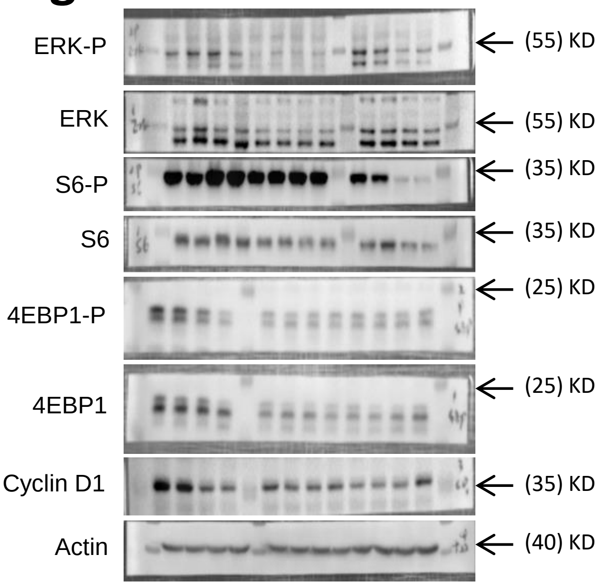

Figure 2e

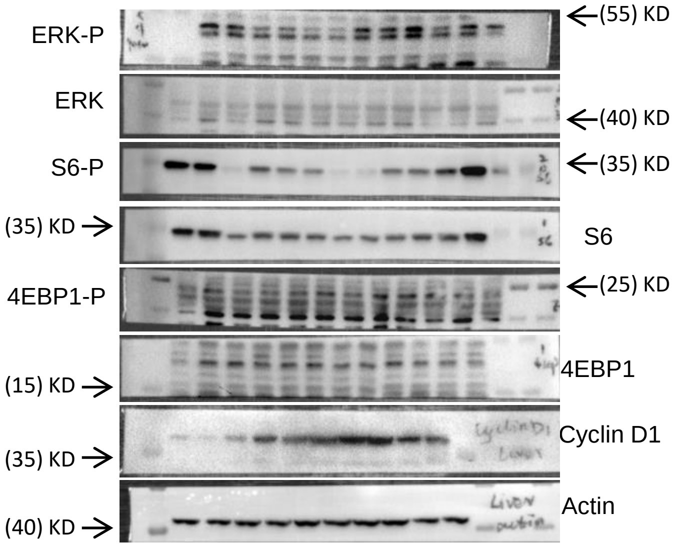

Figure 5b

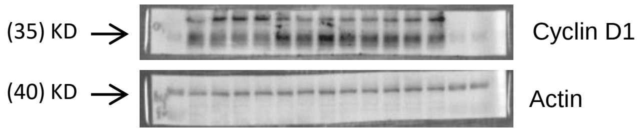

Figure 5f

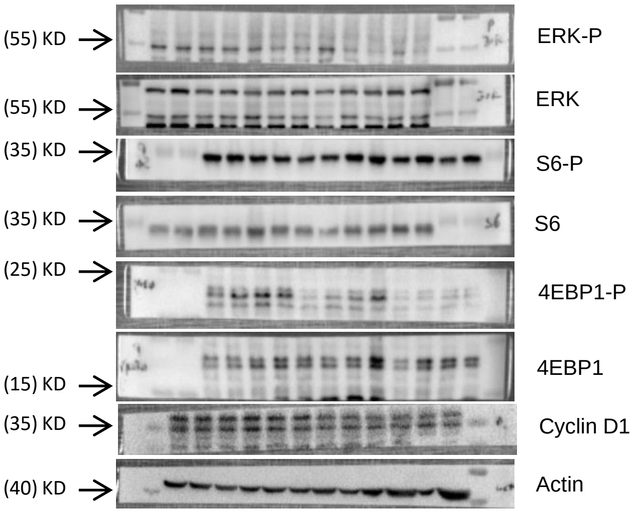

Figure 5h

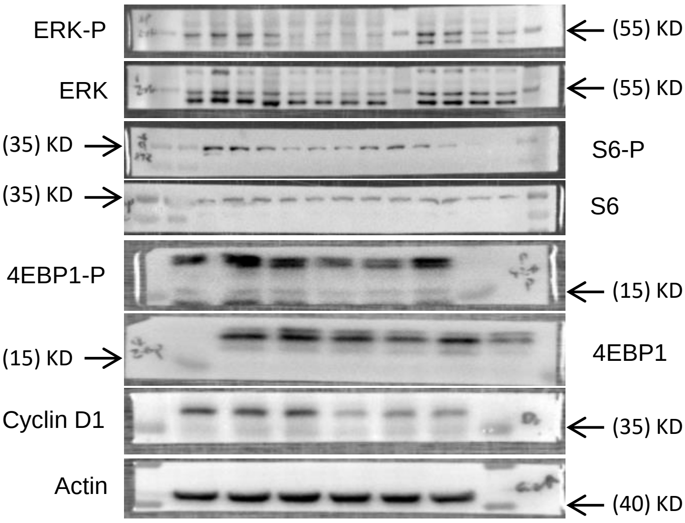

Figure 7f

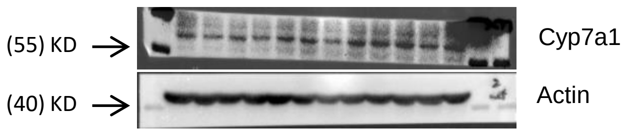

Figure S4b

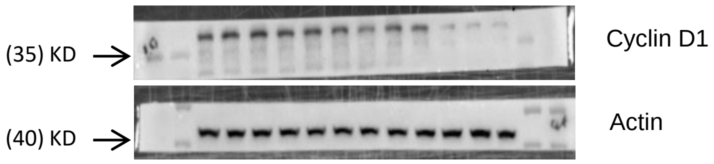

Supplementary Table 1:Primer pair sequences

mice					
Gene	Primer	Sequence	Gene	Primer	Sequence
Actin	Forward	TTCTTTGCAGCTCCTTCG	Tgfβ1	Forward	GCAGTGGCTGAACCAAGGA
	Reverse	TTCTGACCCATTCCCACC		Reverse	AGCAGTGAGCGCTGAATCG
CcnA2	Forward	GTGGTGATTCAAAACTGCCA	Shh	Forward	GAAGGTCTTCTACGTGATCGAG
	Reverse	AGAGTGTGAAGATGCCCTGG		Reverse	TTCAGCCACCACGTACAC
CcnB1	Forward	AAATACCTACAGGGTCGTGAAG	Smo	Forward	TGGCTGACTGGCGGAACTCC
	Reverse	CTCCTGAAGCAGCCTAAATTTC		Reverse	ATCTCTCGGCGGGCACCATC
CcnD1	Forward	AAGCATGCACAGACCTTTGTGG	Fxr	Forward	GCAACCAGTCATGTACAGATTC
	Reverse	TTCAGGCCTTGCATCGCAGC		Reverse	TTATTGAAAATCTCCGCCGAAC
CcnE1	Forward	TCCACGCATGCTGAATTATC	S1pr2	Forward	TCTCTATGCTAAGCACTACGTG
	Reverse	TTGCAAGACCCAGATGAAGA		Reverse	GATGAAAACACCCAGTACGATG
Mki67	Forward	CACAGAGAACAAAGGTGTGAAG	Vdr	Forward	TCAAACTCTGATCTGTACACCC
	Reverse	GGAGACTGCAGAGCTATTTTTG		Reverse	TGGATGCTGTAACTGACAAGAT
α-SMA	Forward	AGAGCTACGAACTGCCTGAC	Fgfr4	Forward	CGTACACAATCTTACGTTGCTT
	Reverse	CCTGTTTTGGCTCCCTATGTCT		Reverse	CAGCGGAATTTGACAGTATTCC
Col1a1	Forward	TGAACGTGGTGTACAAGGTC	Pparα	Forward	GAGCTGCAAGATTCAGAAGAAG
	Reverse	CCATCTTTACCAGGAGAACCAT		Reverse	GAATCTTTCAGGTCGTGTTCAC
Ihh	Forward	GTCCTATGCTCCTCTCACAAG	Nr0b2	Forward	GTCCGACTATTCTGTATGCACT
	Reverse	GATGGAAGGTGCTCTCTTCTAG		Reverse	CTACTGTCTTGGCTAGGACATC
TGR5	Forward	TCAGTCTTGGCCTATGAGCG	Nr1I2	Forward	CGATGTGTCAACCTACATGTTC
	Reverse	CTCGTAGACACCTTTGGGCA		Reverse	CTCAGGATGCACATCTCAAAAG
Cyp7a1	Forward	GGGCAGGCTTGGGAATTTTG	Hgf	Forward	ACCTACAGGAAAACTACTGTCG
	Reverse	AACGCTCAGCAGTCGTTACA		Reverse	TGCATTCAACTTCTGAACACTG
Cyp27a1	Forward	GAGTACGGAGGGTCCAGGAA			
	Reverse	GTCCCAAAGGAGGTTGTCCA			
human					
Actin	Forward	ATTGCCGACAGGATGCAGAA	Ihh	Forward	AACTCGCTGGCTATCTCGGT
	Reverse	ACTCCTGCTTGCTGATCCAC		Reverse	GCCCTCATAATGCAGGGACT
α-SMA	Forward	GGCATTCACGAGACCACCTAC	Col1a1	Forward	CAACGAGATCGAGATCCGC
	Reverse	CGACATGACGTTGTTGGCATAC		Reverse	ACAGGGCCAACGTCGAAG
