# Supplementary material for: miR-182-5p promotes hepatocyte-stellate cell crosstalk to facilitate liver regeneration
Source: Commun Biol. 2022 Aug 1;5:771. doi: 10.1038/s42003-022-03714-0 (PMC9343643; doi:10.1038/s42003-022-03714-0)
Supplement: Supplementary file 3 — Description of Additional Supplementary Files [file 42003_2022_3714_MOESM3_ESM.pdf]

Description of Additional Supplementary Files

File name: Supplementary Data 1
Description: Source data.

File name: Supplementary Data 2
Description: Source data.
