# Supplementary figures and images for: miR-182-5p promotes hepatocyte-stellate cell crosstalk to facilitate liver regeneration
Source: Commun Biol. 2022 Aug 1;5:771. doi: 10.1038/s42003-022-03714-0 (PMC9343643; doi:10.1038/s42003-022-03714-0)

Fig 5c

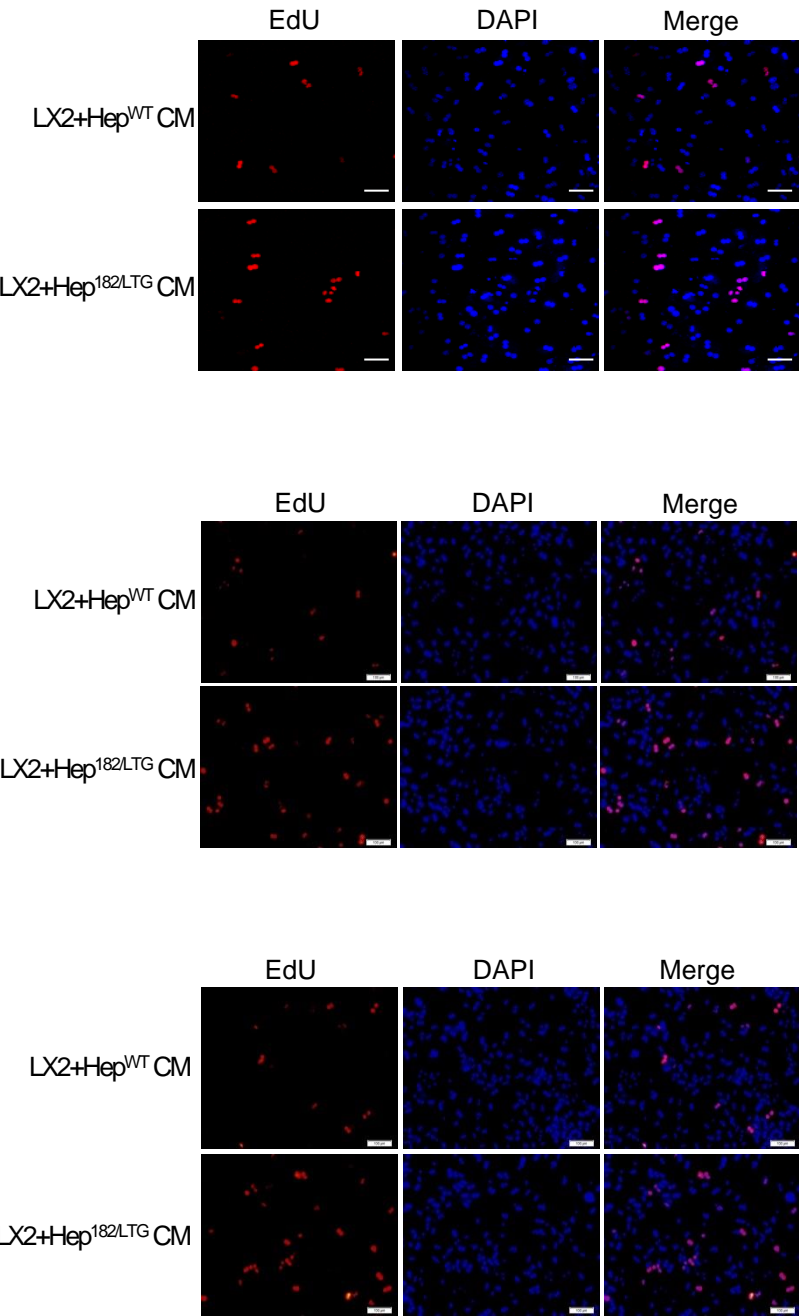

**Fig 5g**

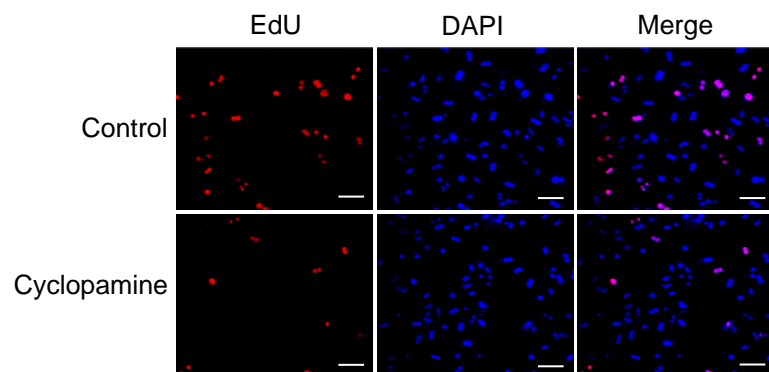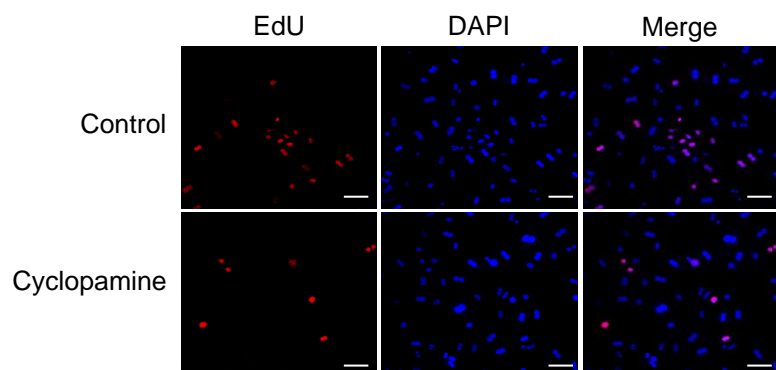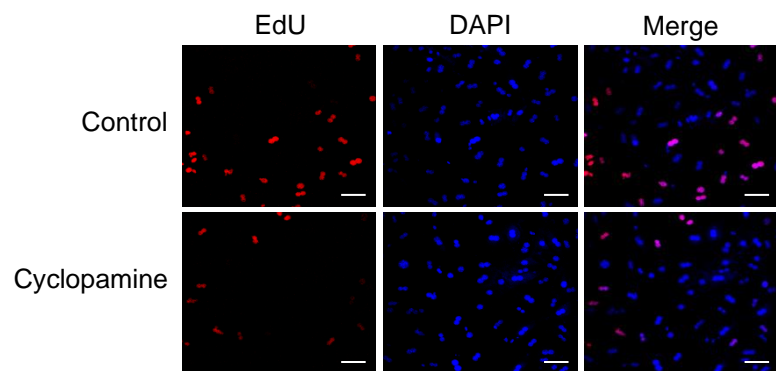

**Fig 5i**

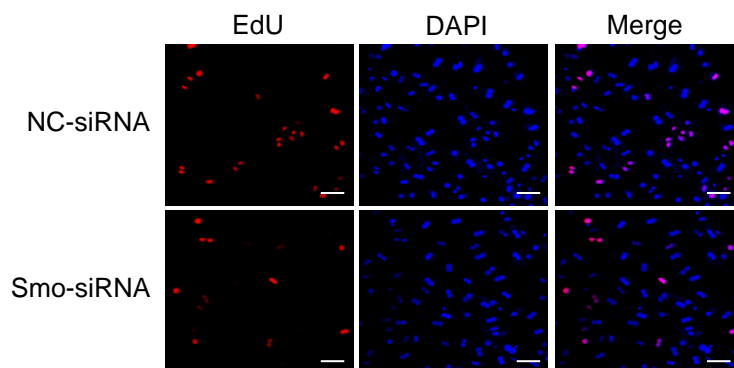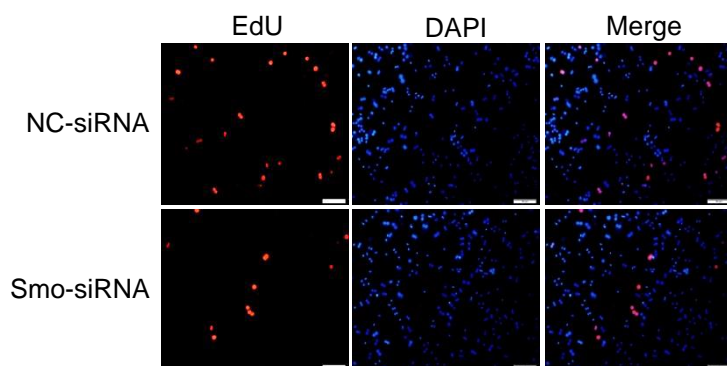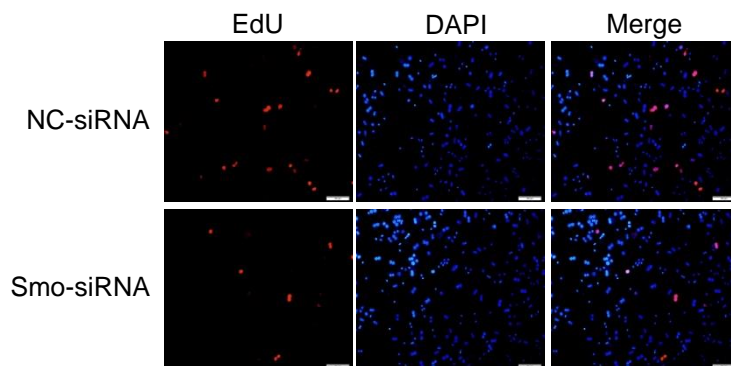

**Fig 7i**

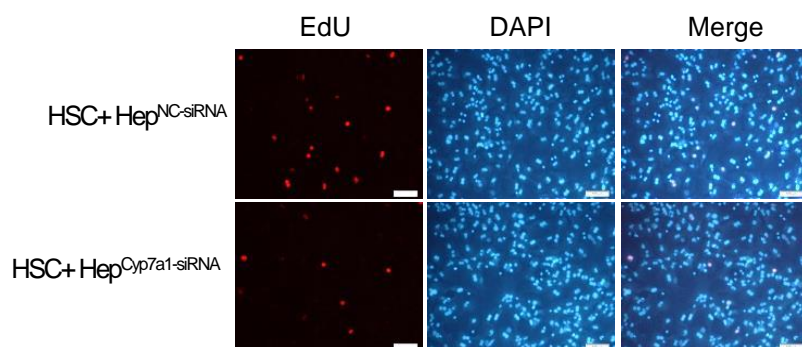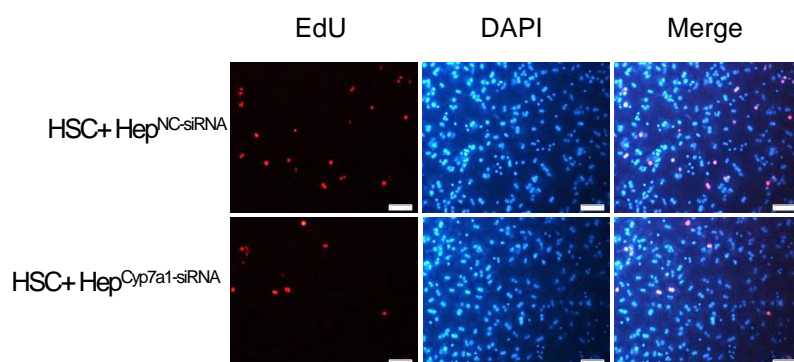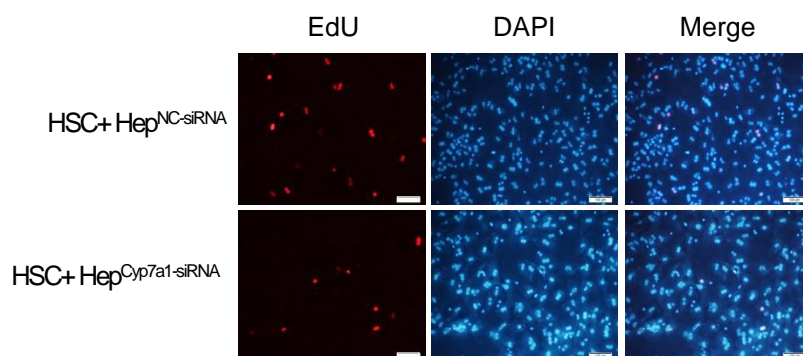

**Fig s2c**

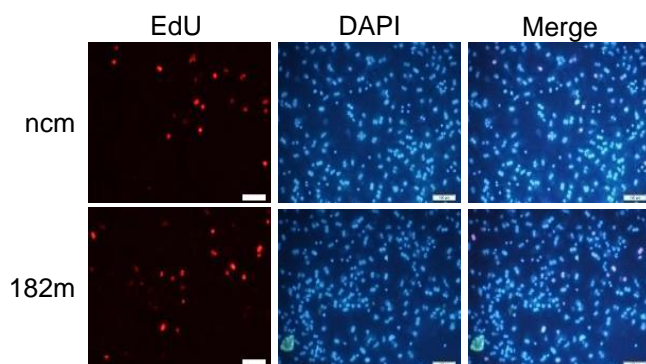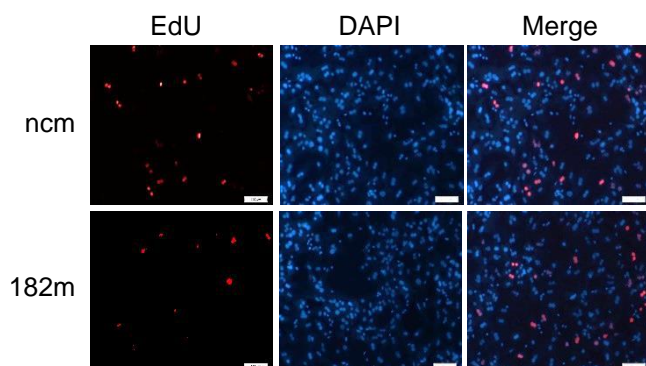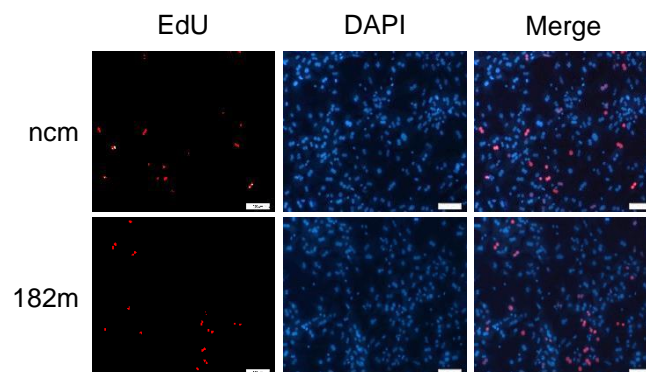

**Fig s2f**

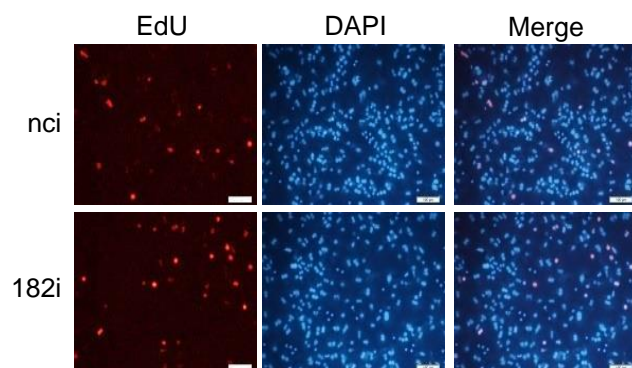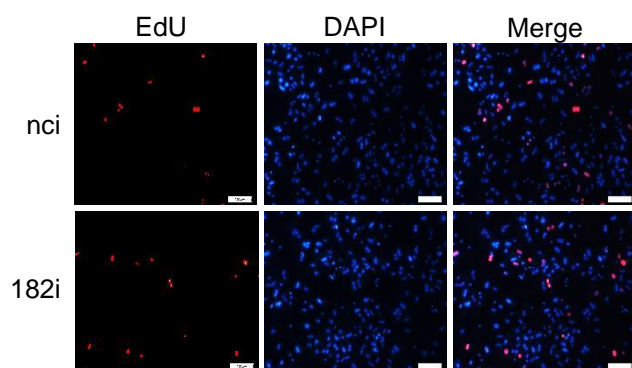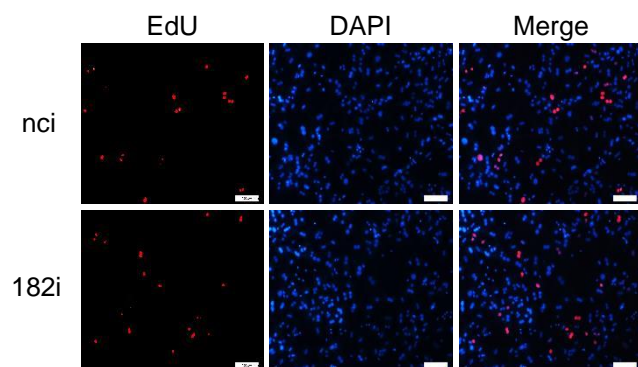

**Fig s4a**

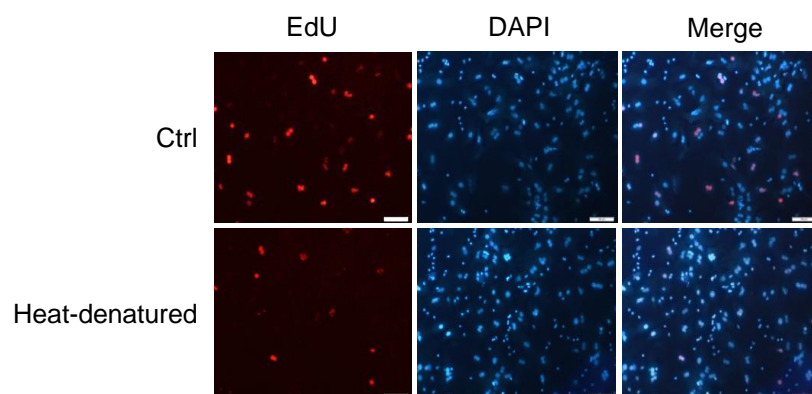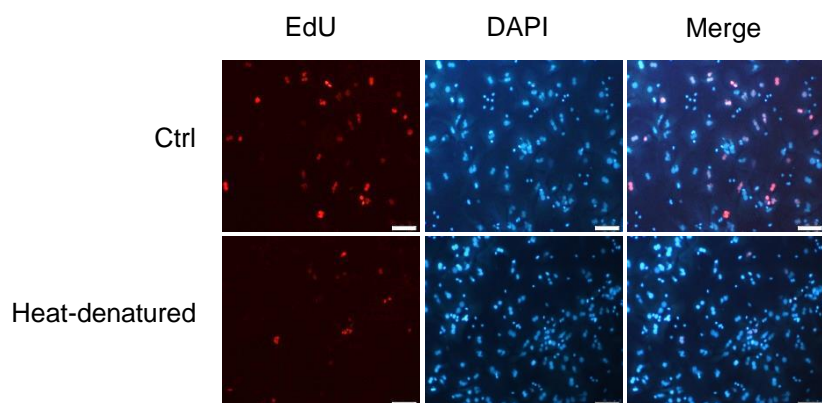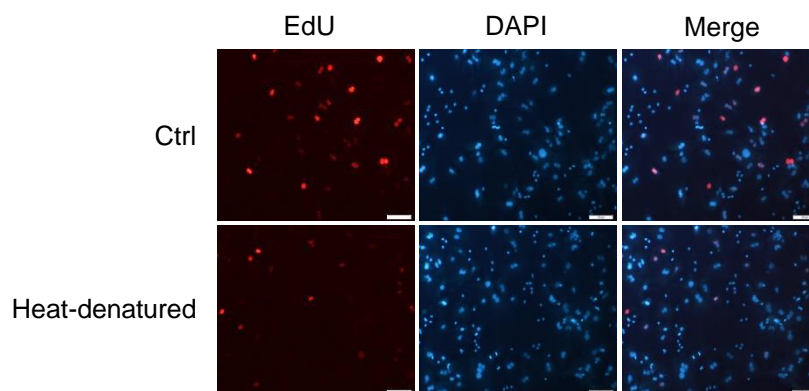

**Fig 1b**

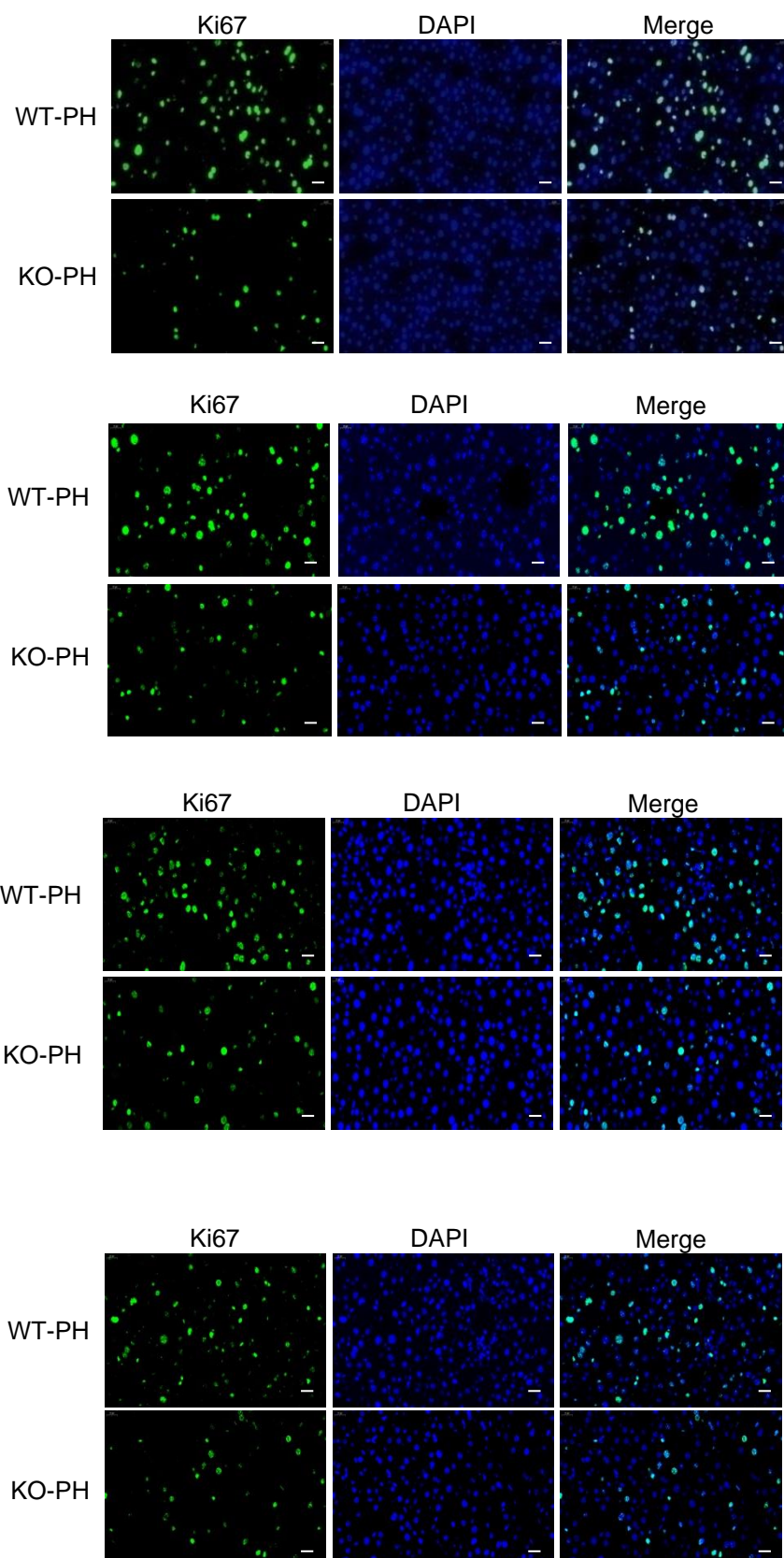

**Fig 2c**

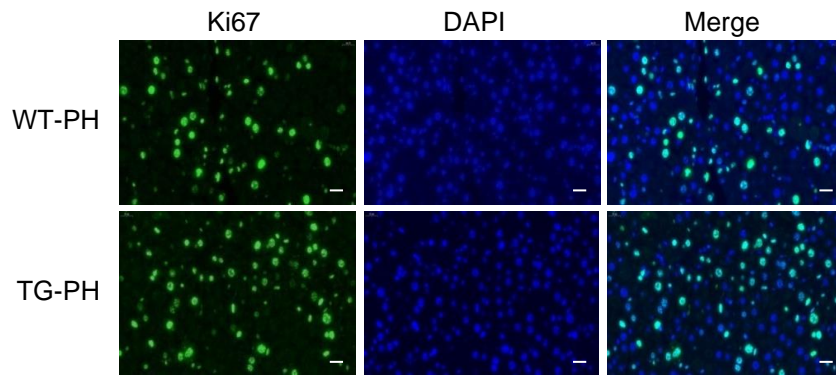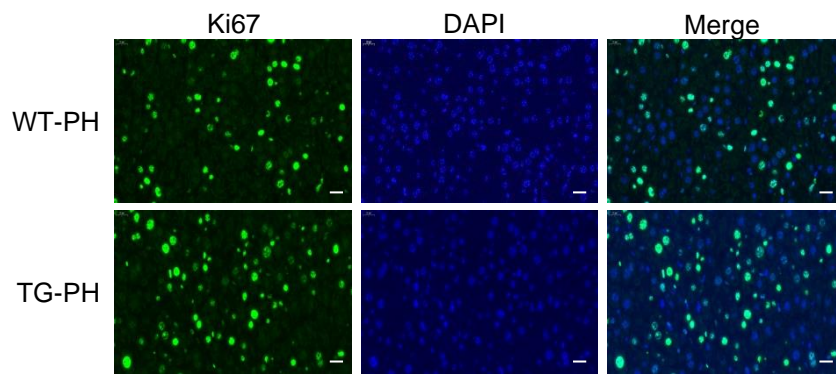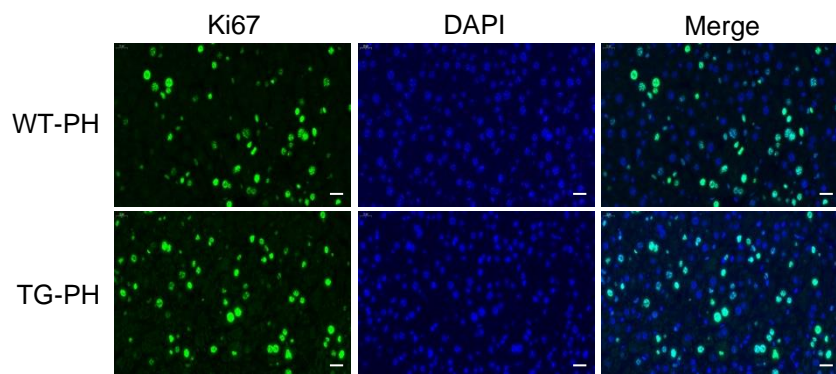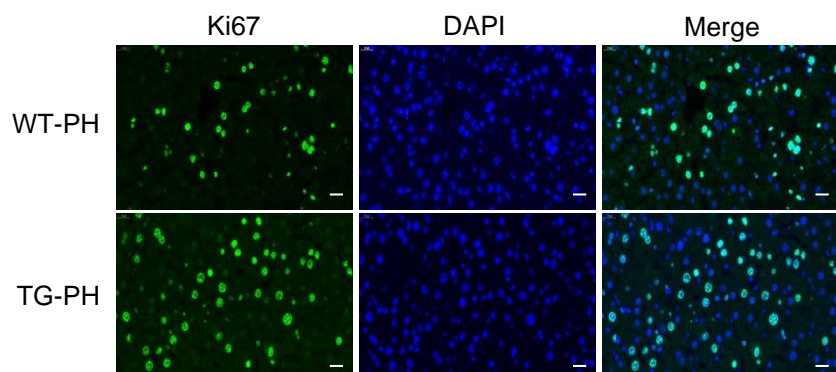

Supplement: Supplementary file 5 — Supplementary Data 2 [file 42003_2022_3714_MOESM5_ESM.pdf]
