# Supplementary material for: miR-182-5p promotes hepatocyte-stellate cell crosstalk to facilitate liver regeneration
Source: Commun Biol. 2022 Aug 1;5:771. doi: 10.1038/s42003-022-03714-0 (PMC9343643; doi:10.1038/s42003-022-03714-0)
Supplement: Supplementary file 6 — Reporting Summary [file 42003_2022_3714_MOESM6_ESM.pdf]

nature portfolio

COMMSBIO-22-0386-B
Corresponding author(s): Dr. Feng Liu, Dr. Wen Meng

Last updated by author(s): Jun 14, 2022

Reporting Summary

Nature Portfolio wishes to improve the reproducibility of the work that we publish. This form provides structure for consistency and transparency in reporting. For further information on Nature Portfolio policies, see our Editorial Policies and the Editorial Policy Checklist.

Statistics

For all statistical analyses, confirm that the following items are present in the figure legend, table legend, main text, or Methods section.

n/a	Confirmed	
☐	☒	The exact sample size (n) for each experimental group/condition, given as a discrete number and unit of measurement
☐	☒	A statement on whether measurements were taken from distinct samples or whether the same sample was measured repeatedly
☐	☒	The statistical test(s) used AND whether they are one- or two-sided<br>*Only common tests should be described solely by name; describe more complex techniques in the Methods section.*
☒	☐	A description of all covariates tested
☒	☐	A description of any assumptions or corrections, such as tests of normality and adjustment for multiple comparisons
☐	☒	A full description of the statistical parameters including central tendency (e.g. means) or other basic estimates (e.g. regression coefficient) AND variation (e.g. standard deviation) or associated estimates of uncertainty (e.g. confidence intervals)
☐	☒	For null hypothesis testing, the test statistic (e.g. F, t, r) with confidence intervals, effect sizes, degrees of freedom and P value noted<br>*Give P values as exact values whenever suitable.*
☒	☐	For Bayesian analysis, information on the choice of priors and Markov chain Monte Carlo settings
☒	☐	For hierarchical and complex designs, identification of the appropriate level for tests and full reporting of outcomes
☒	☐	Estimates of effect sizes (e.g. Cohen's d, Pearson's r), indicating how they were calculated

Our web collection on statistics for biologists contains articles on many of the points above.

Software and code

Policy information about availability of computer code

Data collection	No software was used.
Data analysis	Data analysis was conducted using GraphPad Prism 8.0, Microsoft Excel .

For manuscripts utilizing custom algorithms or software that are central to the research but not yet described in published literature, software must be made available to editors and reviewers. We strongly encourage code deposition in a community repository (e.g. GitHub). See the Nature Portfolio guidelines for submitting code & software for further information.

Data

Policy information about availability of data

All manuscripts must include a data availability statement. This statement should provide the following information, where applicable:

- Accession codes, unique identifiers, or web links for publicly available datasets
- A description of any restrictions on data availability
- For clinical datasets or third party data, please ensure that the statement adheres to our policy

Provide your data availability statement here.

Field-specific reporting

Please select the one below that is the best fit for your research. If you are not sure, read the appropriate sections before making your selection.

☒ Life sciences ☐ Behavioural & social sciences ☐ Ecological, evolutionary & environmental sciences

For a reference copy of the document with all sections, see nature.com/documents/nr-reporting-summary-flat.pdf

Life sciences study design

All studies must disclose on these points even when the disclosure is negative.

Sample size	Animal and cell n number was indicated in each figures.
Data exclusions	No data were excluded.
Replication	All attempts at replication were successful.
Randomization	not applicable
Blinding	not applicable

Reporting for specific materials, systems and methods

We require information from authors about some types of materials, experimental systems and methods used in many studies. Here, indicate whether each material, system or method listed is relevant to your study. If you are not sure if a list item applies to your research, read the appropriate section before selecting a response.

Materials & experimental systems

n/a	Involved in the study	
☐	☒	Antibodies
☐	☒	Eukaryotic cell lines
☒	☐	Palaeontology and archaeology
☐	☒	Animals and other organisms
☒	☐	Human research participants
☒	☐	Clinical data
☒	☐	Dual use research of concern

Methods

n/a	Involved in the study	
☒	☐	ChIP-seq
☒	☐	Flow cytometry
☒	☐	MRI-based neuroimaging

Antibodies

Antibodies used	cyclins D1 (Cat#2922),ERK-P (Cat#4377), S6 (Cat#2217), S6-P Cat#2215), 4EBP1 (Cat#9452) and 4EBP1-P (Cat#9459) were from Cell Signaling Technologies, and Cyp7a1(Cat# DF2612) were from Affinity Biosciences. The anti-β-Actin antibody (Cat#A3854) was from Sigma.
Validation	The validation of commercial antibodies used in this study were posted in manufacturer's website.

Eukaryotic cell lines

Policy information about cell lines

Cell line source(s)	LX-2 were purchased from ATCC.
Authentication	LX-2 cell line was not authenticated
Mycoplasma contamination	not contaminated
Commonly misidentified lines (See ICLAC register)	not applicable

Animals and other organisms

Policy information about studies involving animals; ARRIVE guidelines recommended for reporting animal research

Laboratory animals	miR-182-5p+/-, Laboratory Animal Resource Bank, National Institute of Biomedical Innovation, Japan<br>miR-182-5p-Tgflox/flox , Shanghai Model Organisms Center, China
Wild animals	This study did not involved wild animals.
Field-collected samples	The study did not involves samples collected from the field.
Ethics oversight	All animal studies were performed under a protocol approved by the Central South University Animal Care and Use Committee.

Note that full information on the approval of the study protocol must also be provided in the manuscript.
